# The Investigation of Hippocampus-Dependent Cognitive Decline Induced by Anesthesia/Surgery in Mice Through Integrated Behavioral *Z*-Scoring

**DOI:** 10.3389/fnbeh.2019.00282

**Published:** 2020-01-22

**Authors:** Bo Meng, Xiaoyu Li, Bo Lu, Rongjun Liu, Hui Yuan, Xiaojie Zhai, Jinling Qin, Zhang Chen, Jinwei Zheng, Junping Chen

**Affiliations:** ^1^Department of Anesthesiology, HwaMei Hospital, University of Chinese Academy of Sciences, Ningbo, China; ^2^Zhejiang Key Laboratory of Pathophysiology, School of Medicine, Ningbo University, Ningbo, China

**Keywords:** anesthesia, surgery, hippocampus, neuroinflammation, integrated *Z*-score

## Abstract

**Objective:**

Patients undergoing major surgeries may experience certain cognitive decline, which is known as postoperative delirium (POD) or postoperative cognitive dysfunction (POCD). We employed integrated behavioral *Z-*scoring introduced by Guilloux et al. (2011) to investigate the effects of fracture fixation under anesthesia on hippocampus-dependent memory in mice.

**Methods:**

ICR mice (12–14 months) underwent stabilized tibial fracture operation under sevoflurane anesthesia. They were subjected to a battery of successive hippocampus-dependent tests following surgery, including open field test (OF), novel object recognition (NOR), fear conditioning test (FC), and Morris water maze (MWM). The integrated behavioral *Z-*scoring was applied to assess the hippocampus-dependent memory after anesthesia/surgery, and the association between the integrated behavioral *Z*-scores and hippocampal pro-inflammatory cytokines was explored.

**Results:**

Mice after anesthesia/surgery were found to have impaired hippocampus-dependent memory in NOR, FC, and MWM but with different degrees in these aspects as represented by *P-*value and effect size. The integrated memory *Z*-scores based on principal parameters of the above three tests can reduced the variability and increase the comprehensiveness of behavioral results. However, we found no statistic associations between hippocampal pro-inflammatory cytokines and the integrated *Z*-scores, as the elevated cytokines quickly return to normal on postoperative day 3 and/or day 7.

**Conclusion:**

The integrated *Z*-score methodology could facilitate the interpretation of the anesthesia/surgery induced cognitive decline in mice and robustly quantify the behavioral phenotyping of hippocampus-dependent memory.

## Introduction

There has been a great interest in the subject of learning, memory, and consciousness related to anesthesia, with an emerging body of findings seemingly contradictory. Some evidence suggests that both anesthesia and surgery have long-term effects on cognition, while a great deal of clinical and preclinical animal studies showed that postoperative neurocognitive outcomes would not vary with anesthesia methods and anesthetics (Hovens et al., 2014; Silbert et al., 2014) but were impacted by the combined action of anesthesia and surgery. The impaired postoperative cognition is ever known as postoperative delirium (POD) or postoperative cognitive dysfunction (POCD), and recently, is recommended to be classified as perioperative neurocognitive disorders (Evered et al., 2018). As an acute confusion state, POD is typically of short duration and potentially reversible but may be associated with long-term decline in activities of daily living (Shi et al., 2019), while POCD refers to longer-lasting cognitive decline and features disturbance of memory, attention, orientation, and information processing, leading to the increase of postoperative mortality and risk of leaving the labor market prematurely (Steinmetz et al., 2009).

In line with these observations in clinical practice, preclinical studies have demonstrated behavioral changes following anesthesia/surgery in rodents. However, some studies merely resort to a single behavioral test to assess the postoperative cognitive outcomes (Terrando et al., 2011; Degos et al., 2013) which may miss out POD due to its transience and volatility and may not necessarily recognize the persistent neurocognitive impairment as manifested in patients. Therefore, more and more researchers tend to apply a combination of behavioral tests to study the effects of anesthesia and surgery on cognition and affection (Feng et al., 2013; Hovens et al., 2013; Peng et al., 2016; Zhang X. et al., 2016; Yang et al., 2017). However, the problem is that closely related behavioral parameters of learning and memory specific to each different test do not necessarily agree within animals and/or across time, which leads to a difficulty in interpreting the results convincingly. Although the cause of behavioral variability is unknown, it might reflect the real progress of cognitive change over time or is reckoned as natural fluctuations over the underlying mean value (Fisher and Bannerman, 2019). Actually, changes of cognition and emotionality are highly variable between individuals and over time, quite distinct from symptoms of the organic diseases. Currently, in clinical practice, POD is diagnosed by a set of variable symptoms, and POCD is detected by a battery of neuropsychological tests (Evered et al., 2018). Given the variability of behavioral manifestation, Guilloux et al. (2011) introduced *Z-*normalization, a methodology generally used in clinical study meta-analyses, to present behavioral results in mice phenotyping, which turned out to be sensitive and reliable. Using this method, multiple ethological variables can be combined to obtain a single integrated *Z*-score that can show an overall description of that behavioral dimension and moreover, can potentially strengthen some susceptible parameters.

Although the pathogenesis of postoperative cognitive impairment is generally considered multifactorial, which remains largely unknown, there is strong evidence showing that neuroinflammation is a key factor in the occurrence and development of neurocognitive disorders. The specific mechanisms include blood brain barrier (BBB) disruption, microglial activation, and increased level of pro-inflammatory cytokines (Terrando et al., 2011; Degos et al., 2013; Hovens et al., 2013, 2014; Zhang X. et al., 2016). Among the pro-inflammatory cytokines, TNF-α (Terrando et al., 2010), IL-6 (Hu et al., 2018), and IL-1β (Cibelli et al., 2010) are three classical indicators of neuroinflammation related to cognitive impairment after surgery. As is well-known, in CNS, the hippocampus is especially sensitive to inflammation (Yirmiya and Goshen, 2011). Thus, most research on anesthesia/surgery induced neuroinflammation focused primarily on hippocampal neuroinflammation as well as hippocampus-dependent learning and memory (Terrando et al., 2011; Degos et al., 2013; Hovens et al., 2013; Zhang X. et al., 2016; Yang et al., 2017). In the present study, we applied the integrated behavioral *Z-*scoring method to investigate hippocampus-dependent ethological results by combining statistical significance and practical importance to analyze the outcomes and provided further insight into the relationship between the levels of pro-inflammatory cytokines and the integrated hippocampus-dependent behavioral *Z*-scores.

## Materials and Methods

### Animals

Male ICR mice (12–14 months, 40–55 g) used in this study were purchased from the Experimental Animal Center of Zhejiang Province, China. All experimental procedures involving animals were approved by the Animal Care and Use Committee of Ningbo University in accordance with the guidelines for the Care and Use of Laboratory Animals by National Institutes of Health (NIH Publications No. 80-23). All animals were fed standard rodent food and water *ad libitum* and were housed (three mice per cage) in a temperature-controlled animal facility with 12-h light/dark cycles.

### Anesthesia and Surgery

Mice were induced with 3–4% sevoflurane in a chamber and maintained with 2–3% sevoflurane-air by mice anesthesia mask during the surgery procedure by using an animal anesthesia device (R500SE, RWD Life Science, Shenzhen China). Anesthesia depth was adjusted by maintaining respiratory rate between 100 and 120 bpm. An open tibial fracture surgery was performed under aseptic conditions as described previously (Terrando et al., 2011; Silbert et al., 2014). Briefly, after being shaved and disinfected, a median incision was performed on the left hind paw. Then, a 0.38-mm pin was inserted in the intramedullary canal, the periosteum was then stripped and osteotomy performed. After producing the intramedullary fixation and fracture, the wound was irrigated with povidone iodine and the skin was sewed with 4/0 Prolene sutures. Thereafter, ropivacaine (1.0% and 0.1 ml) was injected into the subcutaneous tissue of the incision area, and erythromycin ointment was dressed on the wound. Animals were allowed to recover spontaneously from the anesthetic.

### Behavioral Tests

**FIGURE 1:**
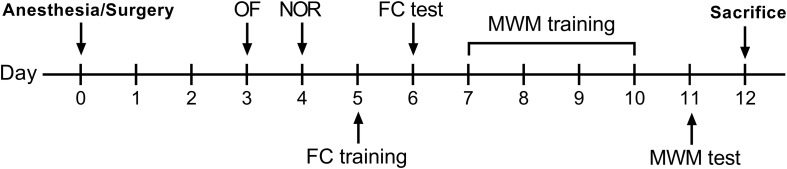
Experimental design. OF, open field; NOR, novel object recognition; FC, fear conditioning; MWM, Morris water maze.

After a 2-day recovery following anesthesia/surgery, behavioral tests were started on postoperative day 3. All tests were conducted in a room adjacent to the housing room with dim light conditions. The experimental design is shown in Figure 1.

#### Open Field

Open field test (OF) was performed to assess the motor activity, exploratory activity, and anxiety-like behavior of the mice. The test was performed on day 3 after surgery. A square (40 × 40 × 40 cm) open field box was divided into a center area (20 × 20 cm) and the rest peripheral area. The mouse was placed in the center of the open field, and the paths were recorded for 5 min and analyzed for total distance moved and time spent in the center using a computerized image analyzing system (ImageMD3000, ZhengHua Biologic Apparatus, Huaibei, China). Time spent in the center area and the percentage of distance moved in the center area were both calculated for scoring the emotionality following anesthesia/surgery.

#### Novel Object Recognition

Novel object recognition test was performed to assess visual and spatial short term memory (Leger et al., 2013). The test was performed on postoperative day 4 using the same apparatus for OF, with two sessions. During familiarization session, the mice facing the wall were placed into the open field box with two identical objects and allowed to explore for 5 min. After a delay of 1 h, the test session was performed for a further 5 min, with one of the familiar objects replaced with a novel object. The paths of the animals were recorded and analyzed with the same computerized image analyzing system (ImageMD3000, ZhengHua Biologic Apparatus, Huaibei, China) on the amount of time and distance taken to explore each object. The recognition index (RI) was calculated according to the following equation: RI = exploring time for novel object/total exploring time for objects. Similarly, in this study, RI of distance was also calculated in view of the fact that motor function might impaired after surgery. Both RI of time and RI of distance were taken as the measure of object or location recognition.

#### Fear Conditioning

Fear conditioning test (FC) is used to assess aversive memory, which is trained to associate with a conditional stimulus (Misane et al., 2005). Freezing behavior is a common and reliable indicator of learning and memory that is measured when subjects are reexposed to the conditional stimulus. The test was conducted using a conditioning chamber (30 × 30 × 45 cm), and the paths and freezing behavior were recorded and analyzed with the matched computerized image analyzing system (SuperFcs, XinRuan Information, Shanghai, China). Mice were trained and tested on separate days according to a similar paradigm that was used as previously described (Zhang et al., 2014; Ni et al., 2018). On postoperative day 5, before training of fear conditioning, the mice were allowed to explore the conditioning chamber for 180 s. The mice were then presented with an auditory cue (70 dB, 3 kH, conditional stimulus) for 30 s, and the unconditional stimulus, 2-s foot-shock (0.75 mA) was administered immediately after termination of the tone. This procedure was repeated with an interval of 60 s, and mice were then removed from the chamber 30 s later. On postoperative day 6, mice were returned into the same chamber where training had occurred, during which no tones or foot-shocks were delivered, to assess recall of contextual fear memory. The percentage of time spent freezing in the contextual fear test was used to score memory and learning abilities. A decrease in the percentage of freezing time indicated impairment of these abilities.

#### Morris Water Maze

Morris water maze (MWM) was performed to assess spatial learning, spatial memory, and cognitive flexibility (Vorhees and Williams, 2006). A water-filled (24–26°C) circular tank 100 cm in diameter and 30 cm deep was surrounded by visual cues and divided into four quadrants. A transparent round platform 10 cm in diameter was placed 0.5 cm below the water surface in quadrant 4 (target quadrant). The water maze protocol started on postoperative day 7 and consisted of 4 days acquisition phase with three massed trials administered each day and a probe trial test following the acquisition phase on postoperative day 11. During the acquisition procedure, the mouse facing the outer edge of the pool was sequentially placed in each quadrant not containing quadrant 4 and allowed to escape to the submerged platform. A trial terminated when the animal reached the platform where it was allowed to remain for 15 s. If the animal failed to find the platform within 60 s, it was manually guided to the platform and left there for 15 s. After each trial, the animal was towel dried and returned to the home cage. The moving paths and time required for locating the hidden escape platform (escape latency) were collected and analyzed with video tracking system (EthoVision XT, Noldus Instruments, Wageningen, Holland). During the probe trial, the platform was removed, and the search pattern of the mice was tracked again. The percentage of time and distance spent in the target quadrant in 60 s were taken as indicators for spatial memory.

### Behavioral *Z*-Score Calculation

To obtain comprehensive and integrated behavioral measures, *Z-*normalization methodology was used in this study. *Z*-score is a dimensionless mathematical tool that allows for mean-normalization of results, and its details and rationale in animal behavior analysis have been described by Guilloux et al. (2011). Briefly, for each behavioral measure, *Z*-score for an individual was calculated using the following equation.

$$Z=\frac{X-\text{μ}}{\text{SD}}$$

*X* represents the individual data for the observed parameter. μ and σ represent the mean and the standard deviation for the control group, respectively. *Z*-score indicates how many standard deviations (SD) an observation (X) is above or below the mean of a control group (μ).

In this study, *Z*-score values were calculated for test parameters measuring emotionality and memory. The directionality of the scores was adjusted so that increased positive *Z*-score values reflected poor performance. To avoid any weighted effect of locomotion on anxious or memorial behavior in the OF, NOR, and MWM, in addition to time data, distance data are also typically used. A single *Z*-score was obtained in OF, NOR, and MWM for each animal by averaging the *Z*-scores of time-related and distance-related parameters, while the OF *Z*-score described the emotionality. Based on a bold hypothesis that the behavioral dimensions of NOR, FC, and MWM were weighed equally, an integrated hippocampus-dependent memory *Z*-score was obtained for each animal based on the three different tests:

$$\mathrm{Hippocampal}\,\mathrm{Memory}\,\mathrm{Score}=\frac{Z_{\text{NOR}}+Z_{\text{FC}}+Z_{\text{MWM}}}{\mathrm{number}\,\mathrm{of}\,\mathrm{tests}}$$

### Enzyme-Linked Immunosorbent Assays

All mice were decapitated under anesthesia with sevoflurane, and brains were quickly removed and dissected to collect the hippocampus. The samples were rinsed with cold saline and homogenized for ELISA assay kits of TNF-α (Cat. No.: EM001; ExCell Bio, Taicang, China), IL-1β (Cat. No.: MTA00B; R&D Systems, Minneapolis, MN, United States), and IL-6 (Cat. No.: EM004; ExCell Bio, Taicang, China). The absorbance was read at 450 nm using a microplate spectrophotometer (Thermo Fisher Scientific Inc., United States). The concentrations were calculated according to the standard curve fitted by four parameter Logistic regression, and values were presented as picogram per milligram. The protocol, reagents preparation, and working standards followed the manufacturer’s instructions (R&D Systems, United States).

### Quantitative Real Time Polymerase Chain Reaction

Total RNA was isolated from homogenized hippocampus using the Trizol Reagent protocol (Life USA). cDNA was synthesized with HiFiScript cDNA Synthesis Kit (ComWin Biotech, Beijing, China). In short, cDNA was amplified by qPCR where a target cDNA (TNF-α, IL-1β, and IL-6) and a reference cDNA (β-actin) were amplified simultaneously using UltraSYBR Mixture Kit (ComWin Biotech, Beijing, China). The amplification was performed at the following conditions: 95°C for 10 min followed by 40 cycles of 95°C for 15 s and 60°C for 30 s. Data were analyzed using the comparative threshold cycle (Ct) method, and all values were expressed relative to the expression of β-actin (2^–Δ^ ^*ct*^).

### Statistical Analysis

All data were expressed as mean ± standard error of the mean (SEM) unless stated otherwise. Analyses were performed using GraphPad Prism 5 (GraphPad Software, San Diego, CA, United States) and compared by unpaired *t-*test and one-way or two-way analysis of variance (ANOVA) followed by Bonferroni’s *post hoc* tests. IBM SPSS Statistics 20 (IBM Corp., Zurich, Switzerland) was used for Pearson’s correlation and analysis of covariance (ANCOVA). The analyzing statistic difference was indicated in the figure legends. *P* < 0.05 was considered as statistically significant.

To estimate the relative magnitude of the differences in the comparisons, Cohen’s *d* effect size coefficients were calculated. The Cohen’s *d* score is defined as the difference between the two means divided by the pooled SD. According to the absolute values of Cohen’s *d* (| d|), the following cutoffs corresponding to the magnitude of the differences were used: | d| ≤ 0.5 for small effect, 0.5 < | d| < 1.0 for moderate effect, 1.0 ≤ | d| < 1.5 for large effect, and | d| ≥ 1.5 for very large effect (Labots et al., 2016).

## Results

### Locomotor Capacity After Anesthesia/Surgery

**FIGURE 2:**
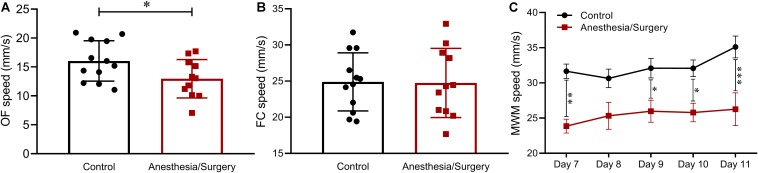
Locomotion across behavioral tests. **(A)** In the OF, the average speed of surgical mice was significantly lower than that of the controls. **(B)** In the FC, there was no significant difference between the two groups. **(C)** In the MWM, the swimming speed of mice after tibial fracture surgery was significantly lower than that of the control. Data are expressed as mean ± SEM (*n* = 11–12) and compared by unpaired *t-*test **(A,B)** or two-way ANOVA followed by Bonferroni’s multiple comparison test **(C)**. ^∗^*P* < 0.05; ^∗∗^*P* < 0.01; ^∗∗∗^*P* < 0.001.

**FIGURE 3:**
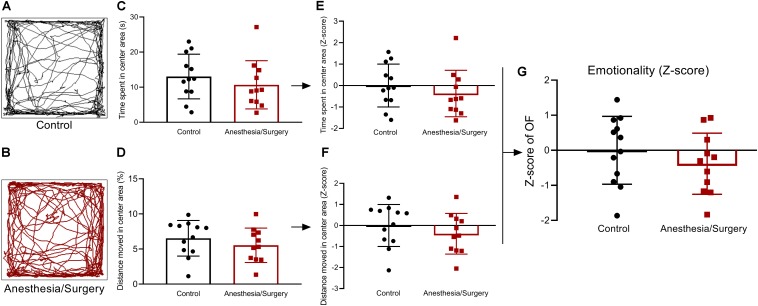
Emotional behavior and emotionality *Z*-scores in OF. **(A,B)** Representative locomotor path of mice in two groups in OF. **(C,D)** No significant difference in the time spent in the center area (s) and distance moved in the center area (%). **(E,F)** Normalization of *Z*-score for the two parameters with the same statistical *P*-values. **(G)** Emotionality *Z*-scores calculated by averaging *Z*-scores of the two OF parameters showed no significant difference between the two groups. Data are expressed as mean ± SEM (*n* = 11–12)and compared by unpaired *t-*test.

One of the mice after anesthesia/surgery was excluded for sciatic nerve injury. Hence, behavioral data of 12 controls and 11 surgical mice was used for analysis. The average moving speed recorded in each behavioral test was used for locomotion analysis. In the OF test on postoperative day 3, surgical mice demonstrated a significant deterioration of ground locomotion compared with controls (Figure 2A) but showed no differences in the FC test on day 5 (Figure 2B). As to the test of MWM, there was a more noticeable decline of swimming ability in mice following tibial fracture surgery than that of the control group (Figure 3C). Accordingly, considering that locomotor activity results varied with different behavioral tests, in subsequent analyses, *Z-*normalization methodology was introduced to reduce statistical bias.

### Emotionality After Anesthesia/Surgery

The emotionality was assessed by OF test (Figures 3A,B), and no significant difference was found in time spent in the center area of OF between two groups (Figure 3C). This result was driven by the fact that there was no significant difference in the percentage of distance moved in the center area (Figure 3D). *Z*-score normalization of the two OF parameters was then performed by transforming absolute values to numbers of SDs above or below the mean of the control (Figures 3E,F). This step yielded, as expected, the same statistical *P*-values as that before normalization. Finally, the *Z*-score values of the two OF parameters per mouse were averaged to obtain a single OF *Z*-score to describe the emotionality, and no significant difference was presented between the two groups (Figure 3G). As a consequence, the anxiety- and depressive-like behaviors were not significantly influenced by surgical trauma.

### Visual Recognition Memory After Anesthesia/Surgery

**FIGURE 4:**
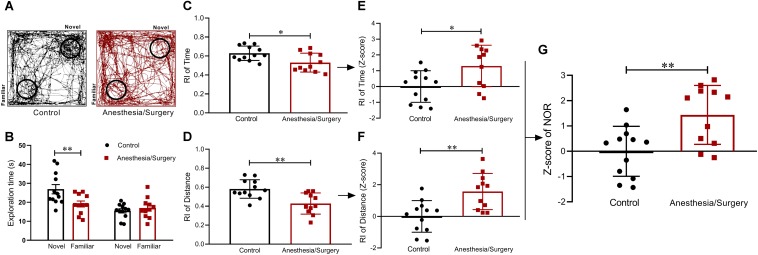
Visual recognition memory behavior and *Z*-scores in NOR. **(A)** Representative exploratory path of two groups of mice in NOR. **(B)** Mice in the control group spent significantly more time in finding the novel object than familiar object, whereas surgical mice did not show a significant difference in finding novel and familiar objects. **(C,D)** RIs of the surgical group based on time and distance were both lower than that of the control. **(E,F)** *Z*-score normalization was performed with the same statistical results as before normalized. **(G)** NOR *Z*-scores obtained by averaging the two RI *Z*-scores indicated that surgical mice performed worse than controls in visual recognition memory. Data are expressed as mean ± SEM (*n* = 11–12)and compared by unpaired *t*-test. ^∗^*P* < 0.05; ^∗∗^*P* < 0.01.

On post-operative day 4, NOR test was performed to assess visual recognition memory (Figure 4A). The mice in the control group were able to discriminate between the familiar and novel objects, whereas the surgical group failed in the test (Figure 4B). The data showed that surgical mice performed worse than controls with a lower RI both on time and distance (Figures 4C,D). Based on these results, *Z*-score transformation was performed for the two NOR parameters, yielding exactly the same statistical *P*-values as before normalized (Figures 4E,F). Afterward, the two normalized *Z*-scores were averaged to obtain a single value per mouse as the NOR *Z*-score. Further analyses of NOR *Z*-scores, which comprehensively reflected the short-term visual recognition memory, suggested that surgical mice performed much worse than controls (Figure 4G).

### Contextual Fear Memory After Anesthesia/Surgery

**FIGURE 5:**
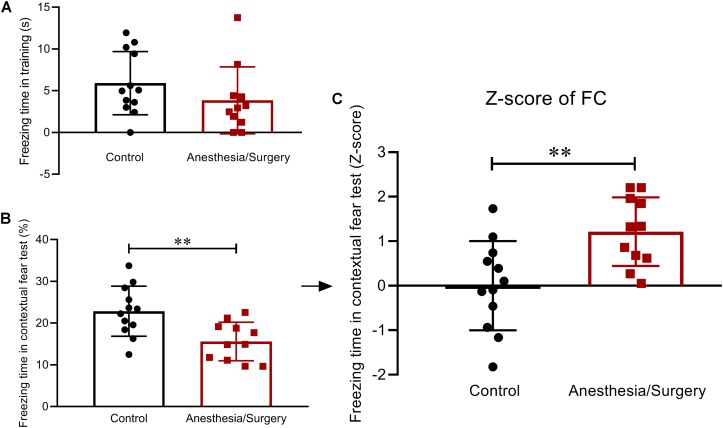
Contextual fear memory behavior and *Z*-scores in FC. **(A)** During the FC training phase, there were no differences in freezing time between the two groups. **(B)** The recall of contextual fear conditioning memories was significantly impaired after anesthesia/surgery. **(C)** The *Z*-scores of freezing time in contextual fear test were calculated and defined as FC *Z*-score. Data are expressed as mean ± SEM (*n* = 11–12)and compared by unpaired *t-*test. ^∗∗^*P* < 0.01.

In the FC, the two groups of mice displayed comparable freezing behavior during the training session on day 5 (Figure 5A). One day later, in the contextual fear test, surgical mice showed a decrease in freezing time (Figure 5B). Then, *Z*-score transformation was performed for freezing time in the test, deservedly, with exactly the same statistical *P*-values as before normalization (Figure 5C). In the following analysis, the *Z*-score of freezing time in the contextual fear test was defined as FC *Z*-score (Figure 5C).

### Spatial Memory After Anesthesia/Surgery

**FIGURE 6:**
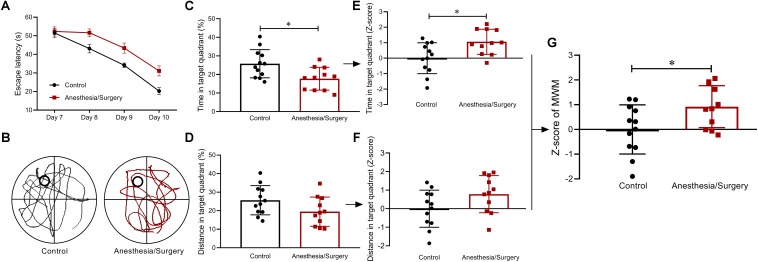
Spatial memory behavior and *Z*-scores in MWM. **(A)** There were no significant differences between groups in escape latency as examined by ANCOVA. **(B)** Representative swimming path of two groups. **(C,D)** Compared with controls, surgical mice spent more time yet with similar distance in target quadrant. **(E,F)** *Z*-score normalization was performed, and similar differences were found between groups as before normalization. **(G)** MWM *Z*-scores were obtained by averaging the two ratios of *Z*-scores, and it showed surgical mice performed worse than controls. Data are expressed as mean ± SEM (*n* = 11–12)and compared by ANCOVA (A) or unpaired *t-*test **(C-G)**. ^∗^*P* < 0.05.

In the MWM, after acquisition phase, all mice were able to learn the task as indicated by decreased latency to find the hidden platform across trials. In consideration of the significant differences in swimming speed between groups (Figure 2C), ANCOVA was used to examine the effects of the surgical trauma on latency with speed as the covariate and showed that there was no significant difference between groups (Figure 6A). On account of the differences in locomotion between groups, the percentages of time and distance in the target quadrant were combined to analyze the performance in the probe trial. Compared with controls, surgical mice spent more time in the target quadrant (Figure 6C) yet with similar distance (Figure 6D). In the same way, *Z*-scores of the time and distance in the target quadrant were calculated, respectively (Figures 6E,F) and averaged to obtain WMW *Z*-score (Figure 6G). As was shown of the statistical results of the WMW *Z*-score, spatial memories were significantly reduced after anesthesia/surgery.

### Integrated Memory *Z*-Scores

**FIGURE 7:**
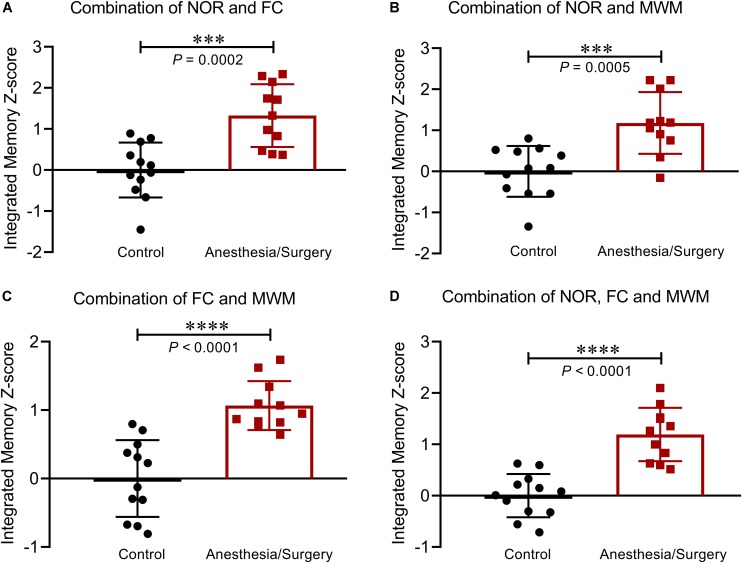
Integrated memory *Z*-scores by different combinations. Integrated hippocampus-dependent memory *Z*-scores were calculated by averaging the *Z*-scores of NOR and FC **(A)**, NOR and MWM **(B)**, FC and MWM **(C)**, and NOR, FC, and MWM **(D)**. Data are expressed as mean ± SEM (*n* = 11–12) and compared by unpaired *t-*test. ^∗∗∗^*P* < 0.001; ^∗∗∗∗^*P* < 0.0001.

Based on the hypothesis that all the three tests are weighted comparably, integrated behavioral *Z-*scoring was employed to investigate the potential of combining the results of NOR and FC (Figure 7A), or NOR and WMW (Figure 7B), or FC and MWM (Figure 7C), or the three of them (Figure 7D) in order to obtain a single evaluation indicator for hippocampus-dependent memory. The analysis of the integrated memory *Z*-scores across different combinations of 2 or 3 behavioral tests revealed that hippocampus-dependent memories were significantly impaired after surgery. Comparing the results of each test using the integrated memory *Z*-scores, we found a more noticeable difference between groups (*P* < 0.001), suggesting that integrated *Z*-scores provide a robust assessment of the effect of surgery on hippocampus-dependent memories.

### Analyses of the Integrated *Z*-Scores

**FIGURE 8:**
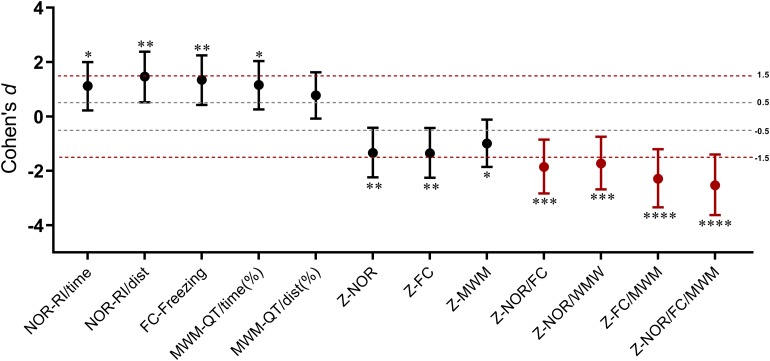
Cohen’s *d* values for behavioral parameters, single test *Z*-scores, and integrated memory *Z*-scores. Error bars represent 95% CI of Cohen’s *d*. ^∗^*P* < 0.05; ^∗∗^*P* < 0.01; ^∗∗∗^*P* < 0.001; ^∗∗∗∗^*P* < 0.0001 for the statistical significance in the corresponding unpaired *t*-test.

As a single indicator, the integrated memory *Z*-score summarized the performances across different behavioral tests (Table 1). It is noticeable that the integrated test or memory *Z*-scores are based on that the SD of *Z-*normalization values are similar across parameters and tests, which means averaging *Z*-values should avoid weighted effects of one parameter or one test over another. The similar weight between the two parameters of NOR and MWM test was verified by the significance of the Pearson’s correlation coefficients (*P* < 0.01) (Table 1). For the integrated memory *Z*-score, the similar weight of NOR, FC, and MWM could be explained by their similar statistical results between groups at a certain extent (Table 1), which could be further confirmed by the consistent large effect sizes (Figure 8). The integrated memory *Z*-scores augmented the statistical significance (Table 1, *P* ≤ 0.0005 in integrated memory *Z*-scores *versus* 0.004 ≤ *P* ≤ 0.03 in single test *Z*-scores, in the *t-*test), while the effect sizes were advanced from “large effect” in single test to “very large effect” in the integrated memory *Z*-scores (Figure 8).

**TABLE 1:** Correlations between the behavioral parameters and *Z*-scores, and the *P*-values for unpaired *t*-test between groups, corresponding to Figures 4–7.

Parameter, *Z*-score		NOR test		FC test	MWM test		*t-*test	Figures
		RI/time	RI/dist	Context	QT/time(%)	QT/dist(%)	*P*-value	
Parameters of test	NOR-RI/time	–	–	–	–	–	0.0137	Figure 4C
	NOR-RI/dist	0.895**	–	–	–	–	0.0021	Figure 4D
	FC-Context	0.343	0.358	–	–	–	0.0040	Figure 5C
	MWM-QT/time(%)	0.155	0.239	0.000	–	–	0.0113	Figure 6C
	MWM-QT/dist(%)	0.190	0.210	–0.130	0.894**	–	0.0754	Figure 6D
Single Test *Z*-score	Z-NOR	–0.973**	–0.974**	–0.360	–0.203	–0.205	0.0043	Figure 4G
	Z-FC	–0.343	–0.358	–1.000**	0.000	0.130	0.0040	Figure 5C
	Z-MWM	–0.177	–0.231	0.067	–0.973**	–0.973**	0.0272	Figure 6G
Integrated Memory *Z*-score	Z-NOR/FC	–0.830**	–0.839**	–0.789**	–0.133	−0.063*	0.0002	Figure 7A
	Z-NOR/WMW	–0.794**	–0.825**	0.218	–0.697**	–0.699**	0.0005	Figure 7B
	Z-FC/MWM	–0.384	−0.433*	–0.702**	–0.694**	–0.597**	<0.0001	Figure 7C
	Z-NOR/FC/MWM	–0.786**	–0.817**	–0.641**	–0.548**	−0.488*	<0.0001	Figure 7D

RI/time, recognition index based on time for NOR test; RI/dist, recognition index based on distance for NOR test; Context, percentage of freezing time in contextual FC test; QT/time(%), percentage of swimming time in target quadrant for MWM probe trail; QT/dist(%), percentage of swimming distance in target quadrant for MWM probe trail. Data are expressed as Pearson’s correlation coefficient R. *, correlation is significant at the 0.05 level; **, correlation is significant at the 0.01 level.

### Hippocampal Inflammation Following Behavioral Tests After Anesthesia/Surgery

**FIGURE 9:**
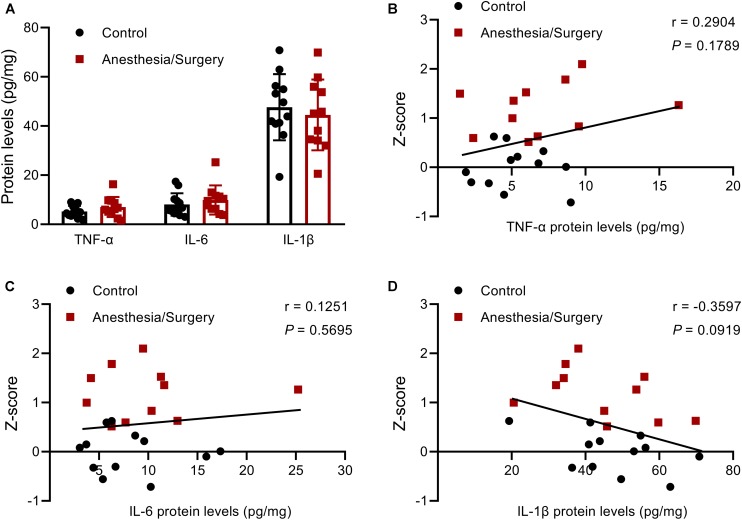
Hippocampal pro-inflammatory cytokines levels, and their relationship with the integrated memory *Z*-scores. **(A)** There were no significant differences between groups in TNF-α, IL-6, and IL-1β levels in the hippocampus on postoperative day 12. **(B–D)** There were no significant correlations between the three pro-inflammatory cytokines levels and the integrated memory *Z*-scores calculated from NOR, FC, and MWM. Data are expressed as mean ± SEM (*n* = 11–12) **(A)** or *Z*-score values corresponding to pro-inflammatory cytokines levels **(B–D)**. Data are analyzed by unpaired *t*-test **(A)** or Pearson’s correlation **(B–D)**.

After all the behavioral tests were finished, on postoperative day 12, the mice of the two groups were sacrificed, and the hippocampi were harvested for ELISA assays of TNF-α, IL-6, and IL-1β. The results showed that there were no significant differences in the three pro-inflammatory cytokines between groups (Figure 9A). Some studies reported that there was an inverse relationship between levels of pro-inflammatory cytokines and behavioral performance after surgery (Kawano et al., 2015; Locatelli et al., 2018). Accordingly, we decided to investigate the correlation between three hippocampal cytokine levels and the integrated memory *Z*-scores by combinations of different behavioral tests. However, no significant correlation was found (Figures 9B–D). But it should be noticed that there might be a certain inverse relationship between IL-1β levels and the integrated memory *Z*-scores (*P* < 0.1) (Figure 9D).

### Hippocampal Inflammation at 24 h After Anesthesia/Surgery

**FIGURE 10:**
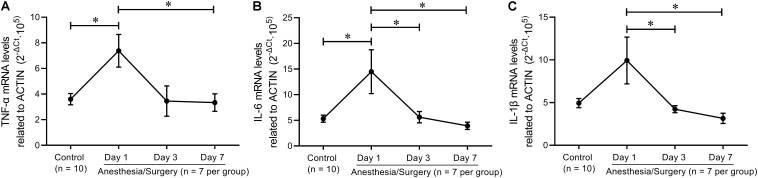
Hippocampal inflammatory trend after tibial fracture surgery. **(A)** TNF-α mRNA levels were significantly increased on day 1 and returned to control levels on day 7. **(B)** IL-6 mRNA levels on days 1, 3, and 7 were higher than that of controls. **(C)** IL−1β mRNA levels on day 1 were higher than that on day 3 and day 7. Data are expressed as mean ± SEM (*n* = 7–10) and compared by one-way ANOVA followed by Bonferroni’s multiple comparison test. ^∗^*P* < 0.05.

It was revealed that hippocampal levels of TNF-α, IL-6, and IL-1β were significantly increased at 24 h, but not on day 7 after abdominal surgery (Zhang Z. et al., 2016). Similarly, carotid artery exposure was reported to induce elevation of hippocampal levels of TNF-α, IL-6, and IL-1β in 4 days, other than 12 days, after surgery (Wang et al., 2017). In order to verify these findings in 12–014 months mice after tibial fracture surgery, we investigated hippocampal pro-inflammatory cytokine mRNA levels on postoperative day 1, day 3, and day 7, respectively, almost all of which were significantly increased on day 1 and decreased rapidly close to control levels on day 3 and/or day 7 (Figures 10A–C).

## Discussion

In recent decade, more and more studies resort to different types of surgical trauma to induce cognitive decline in rodents, aiming to reveal the underlying mechanisms of POCD and POD. Among these types of surgical trauma, open tibial fracture with intramedullary fixation (Cibelli et al., 2010; Terrando et al., 2010, 2011; Degos et al., 2013; Hu et al., 2018; Ni et al., 2018) and exploratory laparotomy (Hovens et al., 2013, 2014; Zhang Z. et al., 2016; Huang et al., 2018; Zhang et al., 2019) are most frequently used. Compared with exploratory laparotomy, we found that tibial fracture surgery was more suitable for models of POCD and POD in mice, considering its higher survival rate and more standardized procedure (unpublished data). Besides, considering that motor activity was not significantly changed after tibial fracture surgery in most studies (Cibelli et al., 2010; Terrando et al., 2010, 2011; Degos et al., 2013; Hu et al., 2018; Ni et al., 2018), we decided to apply this trauma model in the present study. Unfortunately, however, we found that motor activity was actually affected after tibial surgery (Figure 2), which might be attributed to the higher age of the mice we used (12–14 months) while the studies mentioned above used adult rodents around 3 months. To solve this problem here, *Z-*normalization, a methodology that could reduce statistical bias, was applied across hippocampus-dependent memory tests by combining time- and distance-related parameters. In addition, behavioral variability was obvious in the present study as demonstrated by the different levels of statistical significance of behavioral parameters from the principal component analyses (PCA) (Table 1). Hence, we employed integrated memory *Z*-score in order to provide an intuitive and straightforward interpretation of the behavioral tests. Furthermore, we tried to demonstrate the relationship between pro-inflammatory cytokines and the integrated behavioral *Z*-scores but found no statistically significant associations (Figure 9). This, as we believed, might be attributed to the fact that elevated pro-inflammatory cytokines decreased almost to the baseline on postoperative day 3 and/or day 7 (Figure 10).

In clinical practice, POD is frequently diagnosed by confusion assessment method (CAM) including a set of variable symptoms, such as inattention and disorganized thinking, while POCD manifests persisting cognitive decline and is present over several, cognitive function decline and, with large inter-individual differences (Newman et al., 2007; Evered et al., 2018). Considered as age-related neurocognitive disorders, however, POD or POCD may not be featured with perceptible cognitive impairment, quite distinct from the typical syndrome of dementia in Alzheimer’s disease. At present, POCD could be only diagnosed by a battery of neuropsychological tests. For those reasons, researchers in this field tend to employ behavioral test battery to investigate anesthesia/surgery induced cognitive changes in animals (Feng et al., 2013; Hovens et al., 2014; Peng et al., 2016; Zhang X. et al., 2016; Yang et al., 2017). Among animal studies, four behavioral tests, OF, NOR, FC, and MWM, were frequently used for assessing learning and memory and hence, were chosen in the present study and arranged to form a test battery in an order of increasing stress intensity.

In OF, emotional behaviors did not change after tibial fracture surgery, which is consistent with the results of some studies (Feng et al., 2013; Zhang Z. et al., 2016). Hovens et al. (2013, 2014), though, revealed that abdominal surgery could affect anxiety and depressive-like behaviors of 18–20 months rats. The contradictory results might be a result of different surgical trauma applied and/or the different ages of animals as aging plays a vital role in the development of dementia and depression (Araújo et al., 2015). In NOR, a decline in short term visual recognition memory after tibial surgery is shown. Evidence is accumulating that NOR-related memory can be affected by different types of surgical trauma, such as abdominal surgery with upper mesenteric artery clamped (Hovens et al., 2014), or laparotomy (Kawano et al., 2015), and tibial fracture surgery (Vacas et al., 2017). Also, in the FC test, contextual-dependent memory was impaired after anesthesia/surgery as demonstrated by Terrando et al. (2010, 2011), which was found in the learning phase. But, it is of note that Terrando et al. preformed FC training paradigm before surgery specifically for investigating the storage of memory, while we preformed it after surgery for investigating both the encoding and storage of memory, similar to other studies (Hovens et al., 2013; Zhang et al., 2014; Zhang X. et al., 2016). As to MWM, there was no significant difference in the latency across the training phase between groups. With FC results considered together, we believe that surgical trauma does not affect learning process as proved by other studies (Wang et al., 2016; Zhang et al., 2019).

Could postoperative “chronic stress” just like pain, infection, sleep disorder and so on, other than anesthesia/surgery itself, be a major factor leading to POCD and POD? Unfortunately, so far, aging and major surgical trauma are the only two most acknowledged independent risk factors for POCD and POD as indicated by numerous clinical trials (Newman et al., 2007; Evered et al., 2018). Accordingly, in preclinical studies, these two factors are the main elements to mimic POCD and POD in animals. No matter what exactly leads to the cognitive decline following anesthesia/surgery, POCD and POD surely existed. Here, therefore, what we focused is on how to easily detect and interpret cognitive decline after anesthesia/surgery in animal models.

Next, in order to facilitate the interpretation of all the above behavioral results obtained from PCA, *Z-*normalization and the integrated *Z-*scoring were introduced in the study. As a commonly applied method in clinical trials, *Z*-score takes into consideration the differences from mean group values in terms of numbers of SD away from the control or baseline mean (Simpson and Fowler, 2018). *Z-*normalization requires that the variables are normally distributed. In this study, the seven parameters of behavioral tests for *Z-*normalization are typical indicators that are generally analyzed with parametric tests in many studies, and all obey normal distribution as verified by both Kolmogorov-Smirnov test and Shapiro-Wilk test (Supplementary Tables 1, 2). There have been several studies employing *Z*-scores to investigate the emotionality of rodents (Guilloux et al., 2011; Labots et al., 2016, 2018). In the present study, we obtained the emotionality *Z*-scores in OF test by averaging the *Z*-scores of time- and distance-related parameters, as locomotor activity was impaired after tibial surgery. And by the same token, the integrated *Z*-scores of both NOR and MWM were calculated in the same way. As a consequence, the integrated behavioral *Z*-scores reduced the behavioral variables measured to a motivational system/behavioral dimension comparable to PCA. And as expected, there were statistically significant associations between the *Z*-score of each test and the corresponding parameters from the PCA (Table 1). What needs to be noticed, however, is that the integrated *Z*-scores is based on similar SDs of *Z-*normalization values across parameters and tests. In other words, averaging *Z*-values should avoid weighting effects of one parameter or one test over another. In this study, the similar weighting effects between the integrated *Z*-scores of OF, NOR, and MWM can be evidenced by the significant Pearson’s correlation (Table 1) and similar effect size (Figure 8). For the integrated memory *Z*-score, the similar weight of NOR, FC, and MWM could be explained by their similar statistical significance (Table 1) and Cohen’s *d* values (Figure 8) but with some variables as proved by the poor correlation between parameter results of the different tests (Table 1).

By decreasing the number of behavioral variables across different tests, the integrated memory *Z*-scores augmented the overall statistical significance of anesthesia/surgery induced cognitive impairment (Table 1, 0.0000 ≤ *P* ≤ 0.0005 versus 0.0021 ≤ *P* ≤ 0.0754 in behavioral parameters), suggesting that integrated *Z*-scores provide a robust assessment (i.e., less sensitive to outlier values) of the memory levels. This effect is distinctly demonstrated by Cohen’s *d* values as it advanced from “large effect” in a single test to “very large effect” in integrated memory *Z*-scores (Figure 8). Notably, the goal of the integrated *Z*-score analyses is not only to increase statistical significance but rather to extract underlying trends out of apparently variable results since it is often difficult to reconcile positive results across tests, especially for some behavioral tests that are subject to known variability (Guilloux et al., 2011). However, the integrated approach does not detract from PCA which may reveal nuances in behavioral changes as described above. The advantage of integrated *Z-*normalization is to address inherent difficulties in “consistent” behavioral phenotyping across tests and time and to obtain summarized results for simply and intuitively presenting results.

Finally, *Z-*normalization within and across different behavioral tests results in a single *Z*-score per mouse which may be seen as a quantitative “diagnosis” of their hippocampus-dependent memory after anesthesia and surgery. Previously, Peng et al. (2016) had calculated an integrated *Z*-score for each mouse from a battery of behavioral tests (buried food test, OF, and Y maze test) to study POD in mice. According to Peng et al. (2016) this integrated *Z*-score reflects a comprehensive manifestation of attention, consciousness, and organized thinking, while the integrated *Z*-score in this study mainly reflects the hippocampus-dependent memory considering that NOR (Barker and Warburton, 2011), contextual FC (Misane et al., 2005), and MWM (Garthe and Kempermann, 2013) are relatively special for measuring hippocampus-dependent learning and memory. Besides, the integrated *Z*-score of Peng et al. (2016) only horizontally integrated the behavioral phenotyping across tests that were completed in 1 day but did not longitudinally integrate the behavioral results across time. In the present study, the integrated memory *Z*-score composited the behavioral results across 11 days after surgery. We found that the cognitive decline in MWM seemed lighter than that in NOR and FC. This result might reflect that the postoperative cognitive decline is gradually recovering over time. As Hovens et al. (2014) revealed, the location recognition of rats decreased in the first 2 weeks after surgery, but restored at 3 weeks.

Based on the single integrated memory *Z*-score from three behavioral tests per mouse, we attempted to demonstrate the relationship between hippocampal pro-inflammatory cytokines and the integrated hippocampus-dependent *Z*-scores. However, no significant correlations were found, other than an inverse relationship between IL-1β levels and *Z*-scores (*P* = 0.0919). Hovens et al. (2014) reported that exploratory behavior, but not learning and memory, was significantly correlated with plasma IL-6 levels 24 h following abdominal surgery, and microglial activation in hippocampal CA1 was significantly correlated with the performance of OF, FC, and Y-maze at 43 days after surgery (Hovens et al., 2013). In addition, other studies also found that there was an inverse relationship between hippocampal TNF-α and IL-1β levels, and NOR performance 7 days after abdominal surgery (Kawano et al., 2015; Locatelli et al., 2018). The reason for the negative results of the correlation analysis in the present study was largely due to the fact that hippocampal pro-inflammatory cytokines had almost decreased to baseline after the behavioral tests was completed (Figure 9A), which was further proved by hippocampal TNF-α, IL-6, and IL-1β mRNA levels that decreased to around control levels on day 3 and/or day 7 following surgery (Figure 10). However, the duration of postoperative neuroinflammation is controversial. It is previously reported that hippocampal TNF-α and/or IL-6 and/or IL-1β levels still increased in day 3 (Chu et al., 2018), day 7 (Jiang et al., 2018), even in 2–3 weeks (Hovens et al., 2014; Huang et al., 2018) following different types of surgery. Contrarily, a few studies showed that hippocampal pro-inflammatory cytokines only increased 1 day after surgery, ignoring the type of surgical trauma (Hovens et al., 2013; Wang et al., 2016; Zhang S. et al., 2016), which is consistent with our findings. Thus, it can be inferred from the above results that elevated hippocampal pro-inflammatory cytokines might be the trigger of POD or POCD, while the persistent cognitive decline might be associated with other abnormal biology indicators, which requires further studies to figure it out.

In conclusion, the current study suggests that using *Z*-score methodology can reduce the variability and increase the comprehensiveness of behavioral results across a battery of tests and over a period of time. Given the results from the hippocampus-dependent memory tests, the integrated behavioral *Z*-score can amplify and quantify the effect of anesthesia/surgery on the targeting encephalic region function. In addition, by using this unique comprehensive behavioral value, the association between behavioral phenotyping and the underlying molecular mechanism can be clearly demonstrated although we found no statistically significant associations between hippocampal pro-inflammatory cytokines and the integrated memory *Z*-scores, which was mainly due to a rapid fall of levels of the elevated pro-inflammatory cytokines back to the baseline 1 week after surgery. Overall, though, we believe that this methodology could be used for further investigation of the possible mechanism related to cognitive decline following surgery, which may probably help bridge the gap between clinical and pre-clinical studies of POD and POCD (Hovens et al., 2012).

## Data Availability Statement

All datasets generated for this study are included in the article/Supplementary Material.

## Ethics Statement

This study was carried out in accordance with the guideline for the Care and Use of Laboratory Animals by the National Institutes of Health (NIH Publications No. 80-23). This study was reviewed and approved by the Animal Care and Use Committee of Ningbo University.

## Author Contributions

All authors listed have made a substantial, direct and intellectual contribution to the work, and approved it for publication.

## Conflict of Interest

The authors declare that the research was conducted in the absence of any commercial or financial relationships that could be construed as a potential conflict of interest.
