# Supplementary material for: The Investigation of Hippocampus-Dependent Cognitive Decline Induced by Anesthesia/Surgery in Mice Through Integrated Behavioral Z-Scoring
Source: Front Behav Neurosci. 2020 Jan 22;13:282. doi: 10.3389/fnbeh.2019.00282 (PMC6987045; doi:10.3389/fnbeh.2019.00282)
Supplement: Supplementary file 1 [file Data_Sheet_1.pdf]

Supplementary Material

SUPPLEMENTAL TABLE 1:

Normal distribution test results of the seven parameters in group Control

Group Control	Kolmogorov-Smirnov		Shapiro-Wilk	
	Statistics	*P* value	Statistics	*P* value
OF-time(s)	0.124	0.200	0.965	0.852
OF-dist(%)	0.220	0.115	0.926	0.337
NOR-RI/time	0.196	0.200	0.925	0.331
NOR-RI/dist	0.145	0.200	0.960	0.784
FC-Context	0.114	0.200	0.993	1.000
MWM-QT/time(%)	0.140	0.200	0.949	0.623
MWM-QT/dist(%)	0.115	0.200	0.970	0.160

OF-time(s), time spent in the center area (s) for OF test; OF-dist(%), the percentage of distance moved in the center area for OF test; NOR-RI/time, recognition index based on time for NOR test; NOR-RI/dist, recognition index based on distance for NOR test; Context, percentage of freezing time in contextual FC test; MWM-QT/time(%), percentage of swimming time in target quadrant for MWM probe trail; MWM-QT/dist(%), percentage of swimming distance in target quadrant for MWM probe trail.

SUPPLEMENTAL TABLE 2:

Normal distribution test results of the seven parameters in group anesthesia/surgery

Group<br>Aesthesia/surgery	Kolmogorov-Smirnov		Shapiro-Wilk	
	Statistics	*P* value	Statistics	*P* value
OF-time(s)	0.218	0.152	0.885	0.120
OF-dist(%)	0.132	0.200	0.980	0.967
NOR-RI/time	0.214	0.172	0.897	0.172
NOR-RI/dist	0.155	0.200	0.929	0.404
FC-Context	0.158	0.200	0.928	0.391
MWM-QT/time(%)	0.190	0.200	0.938	0.498
MWM-QT/dist(%)	0.133	0.200	0.939	0.504

OF-time(s), time spent in the center area (s) for OF test; OF-dist(%), the percentage of distance moved in the center area for OF test; NOR-RI/time, recognition index based on time for NOR test; NOR-RI/dist, recognition index based on distance for NOR test; Context, percentage of freezing time in contextual FC test; MWM-QT/time(%), percentage of swimming time in target quadrant for MWM probe trail; MWM-QT/dist(%), percentage of swimming distance in target quadrant for MWM probe trail.
